# Defining natural species of bacteria: clear-cut genomic boundaries revealed by a turning point in nucleotide sequence divergence

**DOI:** 10.1186/1471-2164-14-489

**Published:** 2013-07-18

**Authors:** Le Tang, Yang Li, Xia Deng, Randal N Johnston, Gui-Rong Liu, Shu-Lin Liu

**Affiliations:** 1 Genomics Research Center (one of The State-Province Key Laboratories of Biomedicine-Pharmaceutics of China), Harbin, China; 2 HMU-UCFM Centre for Infection and Genomics, Harbin, China; 3 Department of Biopharmaceutics, Harbin Medical University, 157 Baojian Road, Harbin 150081, China; 4 Departments of Biochemistry and Molecular Biology, Calgary, Canada; 5 Microbiology and Infectious Diseases, University of Calgary, Calgary, Canada

**Keywords:** Natural species, *Salmonella*, Genetic boundary

## Abstract

**Background:**

Bacteria are currently classified into arbitrary species, but whether they actually exist as discrete natural species was unclear. To reveal genomic features that may unambiguously group bacteria into discrete genetic clusters, we carried out systematic genomic comparisons among representative bacteria.

**Results:**

We found that bacteria of *Salmonella* formed tight phylogenetic clusters separated by various genetic distances: whereas over 90% of the approximately four thousand shared genes had completely identical sequences among strains of the same lineage, the percentages dropped sharply to below 50% across the lineages, demonstrating the existence of clear-cut genetic boundaries by a steep turning point in nucleotide sequence divergence. Recombination assays supported the genetic boundary hypothesis, suggesting that genetic barriers had been formed between bacteria of even very closely related lineages. We found similar situations in bacteria of *Yersinia* and *Staphylococcus*.

**Conclusions:**

Bacteria are genetically isolated into discrete clusters equivalent to natural species.

## Background

Bacteria are classified into species, which are organized into higher taxonomic ranks such as genera, families, orders, etc., based on levels of similarity among them. However, the definition of the fundamental taxonomic unit, the species, is still an unsolved issue. Over the past three centuries since their discovery, bacteria have been classified in numerous ways based on morphological, serological, biochemical or genetic properties, with the species being defined differently according to the method used for the classification. As a result, a bacterial pathogen may at one time be defined as an independent species or at another time as a variant of a species along with many other bacteria that share phenotypic or genetic similarities. For example, the human typhoid agent was originally treated as a species with a Latinized scientific name *Salmonella typhi* but later was re-classified as merely a serovar of another species, *Salmonella enterica*, together with over 2000 other “serovars” [1-3]; these 2000 plus serovars are mostly mild or non-pathogenic to humans and were, like *S. typhi*, initially also classified as separate species. Inclusion of the deadly human pathogen *S. typhi* in a species together with thousands of pathogenically very different bacteria has in fact caused enormous confusions in the clinical as well as basic research settings. In addition to medicine, the recognition of natural bacterial species is also important for research and applications in industrial and agricultural areas. Essentially, all such confusions have resulted from the lack of theory-based species concept and of objective criteria-supported species definition.

Currently an expedient way is to categorize bacteria into taxonomic species by arbitrary cut-off values at 70% DNA-DNA association and 97% 16S rRNA sequence identity [4,5]. However, since both kinds of data are continuous, the 70% and 97% criteria can hardly assign bacteria into discrete genetic groupings. More seriously, the wide ranges of genomic variation set by the 70% and 97% criteria would unavoidably classify a great diversity of phylogenetically different bacteria into the same species. Therefore, for a stable classification system that truly reflects the evolutionary relationships of bacteria, the basic taxonomic unit, i.e., species, needs to be defined on the basis of objective criteria that can assign bacteria into discrete genetic as well as biological clusters with clear-cut boundaries.

Previous work already suggests that bacteria exist in discrete clusters as demonstrated by their distinct genome structures [6-8] and significantly reduced recombination efficiency among even very closely related bacteria [9], although it has been unclear whether the genetic isolation among the bacteria is “clear-cut”. Based on our earlier findings with *Salmonella*[10-12], we hypothesize that genetic boundaries may exist to isolate bacteria into phylogenetically discrete clusters equivalent to natural species [13]. In this study, we use *Salmonella* as the primary models to explore the hypothesized genetic boundaries. We found sharp genetic distinctness among bacteria of closely related lineages and demonstrated the existence of an abrupt turning point in sequence divergence between any pair of *Salmonella* lineages compared. When we extended the work to other bacteria, including *Yersinia* and *Staphylococcus*, we found similar genetic boundaries. We propose that bacteria circumscribed by the genetic boundary be considered members of a natural species, and bacteria of a natural species should have cohesive genetic and biological attributes.

## Results

### Genomic sequence comparison: high homogeneity and abrupt divergence within and across *Salmonella* lineages as molecular evidence of genetic boundaries

We used *Salmonella* as the primary model in this study mainly for the close genetic relatedness [14,15] and distinct biological properties [16-18] of these bacteria in addition to the extraordinarily large number of lineages available for comparative studies. The serologically defined *Salmonella* types, called serotypes or serovars, may be monophyletic or polyphyletic. Examples of monophyletic *Salmonella* serotypes are those with antigenic formula 9,12:d:- for *S. typhi* and 1,2,12:a:[1,5] for *S. paratyphi* A. On the other hand, many *Salmonella* serotypes are polyphyletic, such as those with antigenic formula 6,7:c:1,5 that actually includes diverse pathogens *S. paratyphi* C, *S. choleraesuis* and *S. typhisuis* or 1,9,12:a:1,5 that can be differentiated into *S. miami* and *S. sendai* by biochemical assays. Even serotypes with the same name may contain multiple “lineages”, such as *S. paratyphi* B (1,4,[5],12:b:1,2), which can be divided into *d*-tartrate positive and negative lineages, with the former infecting a broad range of hosts and causing gastroenteritis and the latter infecting only humans and causing paratyphoid. The *Salmonella* strains compared in this study are either of monophyletic serotypes or representatives of individual lineages of polyphyletic serotypes according to our previous phylogenetic studies of these bacteria [7,8,19-22]. We compared the genomes of twenty six strains from thirteen *Salmonella* lineages (Additional file 1: Table S1), to reveal potentially important genomic differences that may clearly distinguish the lineages on a phylogenetic basis. For this, we first identified genes common to these genomes. We found that all compared *Salmonella* genomes are indeed highly similar: the strains of different lineages share most of their genes, from 79% as between *S. typhi* and *S. pullorum* (3693 of the 4682 genes of *S. pullorum* RKS5078 are in common with genes of *S. typhi* Ty2) to 93% as between *S. gallinarum* and *S. pullorum* (4034 of the 4347 genes of *S. gallinarum* 287/91 are in common with genes of *S. pullorum* RKS5078; Additional file 2: Table S2). Within a lineage, this percentage may be lower or higher than 90% (Additional file 2: Table S2). As the percentage ranges of shared genes inside and across the *Salmonella* lineages are continuous or even overlapping, the hypothesized genetic boundaries among different *Salmonella* lineages were not supported in this regard.

However, when we compared the levels of sequence identity between homologous genes, a drastic distinction stood up conspicuously, forming an acute turning point in sequence divergence between strains of a pair of lineages compared. Whereas within a particular lineage, most of the genes had 100% sequence identity among independent strains, across different lineages the percentages of genes with 100% sequence identity dropped abruptly (Additional file 3: Table S3). With rare exceptions, the percentages of genes with 100% sequence identity were 85% or higher among strains of the same lineage and 12% or lower across the lineages (Additional file 4: Table S4). The exceptions were seen in the comparison of three lineages, including *S. enteritidis, S. gallinarum* and *S. pullorum*, among which about 40% of their homologous genes have 100% sequence identity (Additional file 4: Table S4). Our explanation is that these three pathogens have diverged not long enough to independently accumulate as many mutations. Nevertheless, clear-cut genetic boundaries have already been formed among them, delineating these three close relatives into distinct lineages.

**Figure 1:**
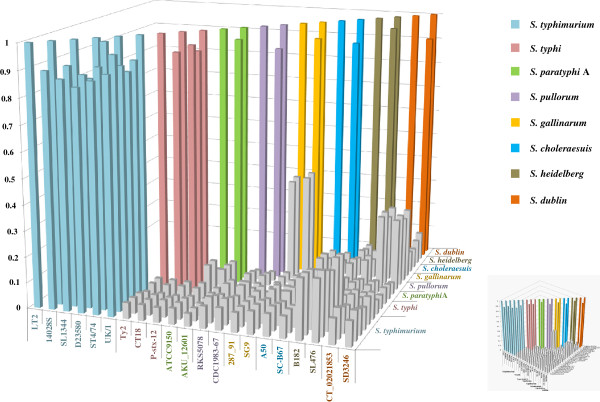
**Genomic comparison among the *Salmonella *strains.** Sequences common to all twenty six strains were concatenated and pair-wise aligned for the number of genes that have 100% sequence identity. The smaller figure at the right lower corner is the main figure viewed from a different point.

The landscape of genomic distinction shown in Figure 1 for the lineages that have two or more strains intuitively demonstrates the existence of genetic boundaries among the *Salmonella* lineages, which prompted us to speculate that genetic barriers may exist to facilitate the formation of genetic boundaries among even very closely related bacterial lineages, such as *S. gallinarum* and *S. pullorum*. We then used this pair of lineages to explore this issue through genomic recombination experiments.

### Genetic barriers assessed by DNA recombination assays between *S. gallinarum* and *S. pullorum*

The fowl pathogens *S. gallinarum* and *S. pullorum* have a common antigenic formula, 1,9,12:-:-, the former causing typhoid and the latter causing pullorum disease (dysentery). They are so closely related that, being originally treated as separate species [15], they have since the mid 1980s been classified into the same serovar of the same species and even the same subspecies (i.e., *S. enterica* subspecies *enterica* Serovar Gallinarum as separate biovars Gallinarum and Pullorum, respectively [3]). However, their biological distinction (causing entirely different diseases) unambiguously tells that they are different organisms (i.e., each being a natural species on its own right). Our recent work also reveals that the two pathogens have accumulated distinct sets of mutations including different pseudogenes [23,24], further demonstrating genetic divergence of the two *Salmonella* lineages. Therefore, the existence of genetic barriers, if experimentally validated, would further support the genetic boundary hypothesis and facilitate the establishment of objective criteria for defining natural species of bacteria. Otherwise, the genetic boundary concept would need reconsideration.

We used the bacteriophage P22 to move DNA between *S. pullorum* and *S. gallinarum* by generalized transduction as previously described [25]. We first moved the Tn*10-*inserted *ompD*159 gene from *S. typhimurium* LT2 [16,26] to four *S. pullorum* strains RKS5078 [23,27], CDC1983-67, SARB51 and 04–6767, and four *S. gallinarum* strains 287/91 [28], RKS5021, SGSC2293 and 91–29327 (see strain information at http://www.ucalgary.ca/~kesander). Then we moved the *ompD*159 gene from one of the eight strains to the other seven strains and repeated this process for all of the eight strains. When we inspected transductants on LB plates containing tetracycline and compared their numbers among the bacterial strains used as the recipients of the DNA carried by the P22 phage, we saw a general tendency in differential efficiency to incorporate the same donor DNA between *S. pullorum* and *S. gallinarum*: transduction of *S. pullorum* recipients with DNA from *S. pullorum* resulted in larger numbers of transductants than with DNA from *S. gallinarum* and, similarly, transduction of *S. gallinarum* recipients with DNA from *S. gallinarum* resulted in larger numbers of transductants than with DNA from *S. pullorum* (Additional file 5: Table S5). To validate this observation and rule out the possibility that a particular genomic DNA segment or a particular bacterial strain might have given non-representative results, we used additional DNA segments (Tn*10*-inserted *leu*-1151, *bio*-102, *oxrA*2 and *cysA*1367, in addition to *ompD*159, which was also included in the second set of transduction experiments for comparisons) and additional *S. pullorum* and *S. gallinarum* strains (Additional file 6: Table S6). Again, the transduction efficiency was lower in across-lineage combinations (i.e., *S. pullorum* or *S. gallinarum* as recipient to receive *S. gallinarum* or *S. pullorum* DNA) than in recipient-donor combinations of the same lineage (Additional file 6: Table S6).

### *Salmonella* lineages as discrete clusters of bacteria: phylogenetic distinction

**Figure 2:**
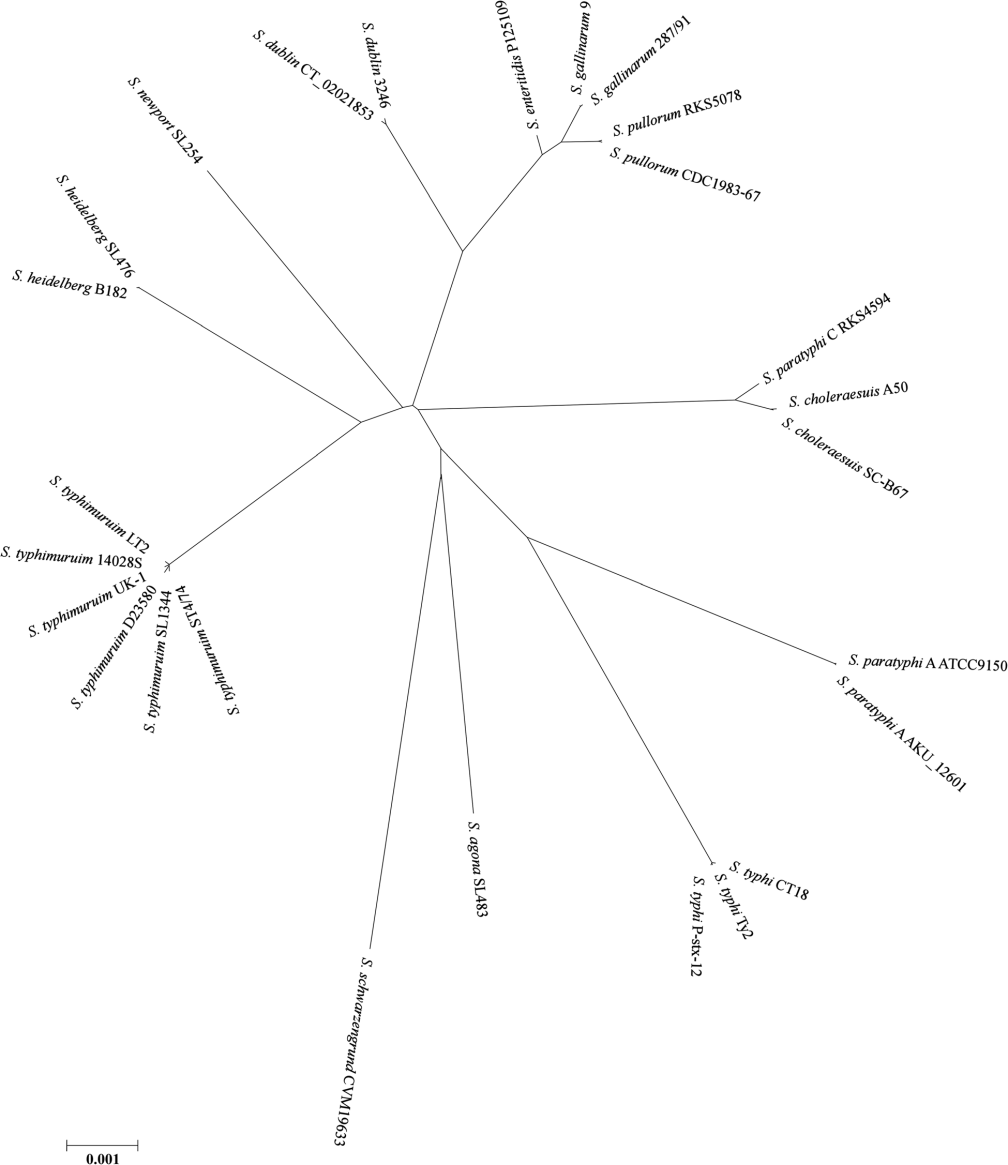
**Phylogenetic tree for twenty six sequenced *Salmonella *strains, based on whole-genome sequences (all conserved regions among the compared genomes are concatenated and aligned for tree construction).**

To further look into the natural relationships of the *Salmonella* lineages, we concatenated the genomic sequences common to all of the 26 *Salmonella* strains and constructed a phylogenetic tree (Figure 2). Remarkably, within the lineages that had two or more strains in our comparison, the strains of the same lineage clustered tightly to a tiny point of a branch on the tree (see *S. typhimurium, S. heidelberg, S. dublin, S. gallinarum, S. pullorum S. choleraesuis, S. paratyphi* A and *S. typhi,* on the tree of Figure 2), further demonstrating the high genetic homogeneity of the bacteria within the same *Salmonella* lineage; conversely, individual lineages are isolated by branches of different lengths, demonstrating the existence of genetic boundaries to circumscribe the bacteria into natural genetic clusters.

### Genome structure comparison of the *Salmonella* lineages: abrupt dissimilarity

**Figure 3:**
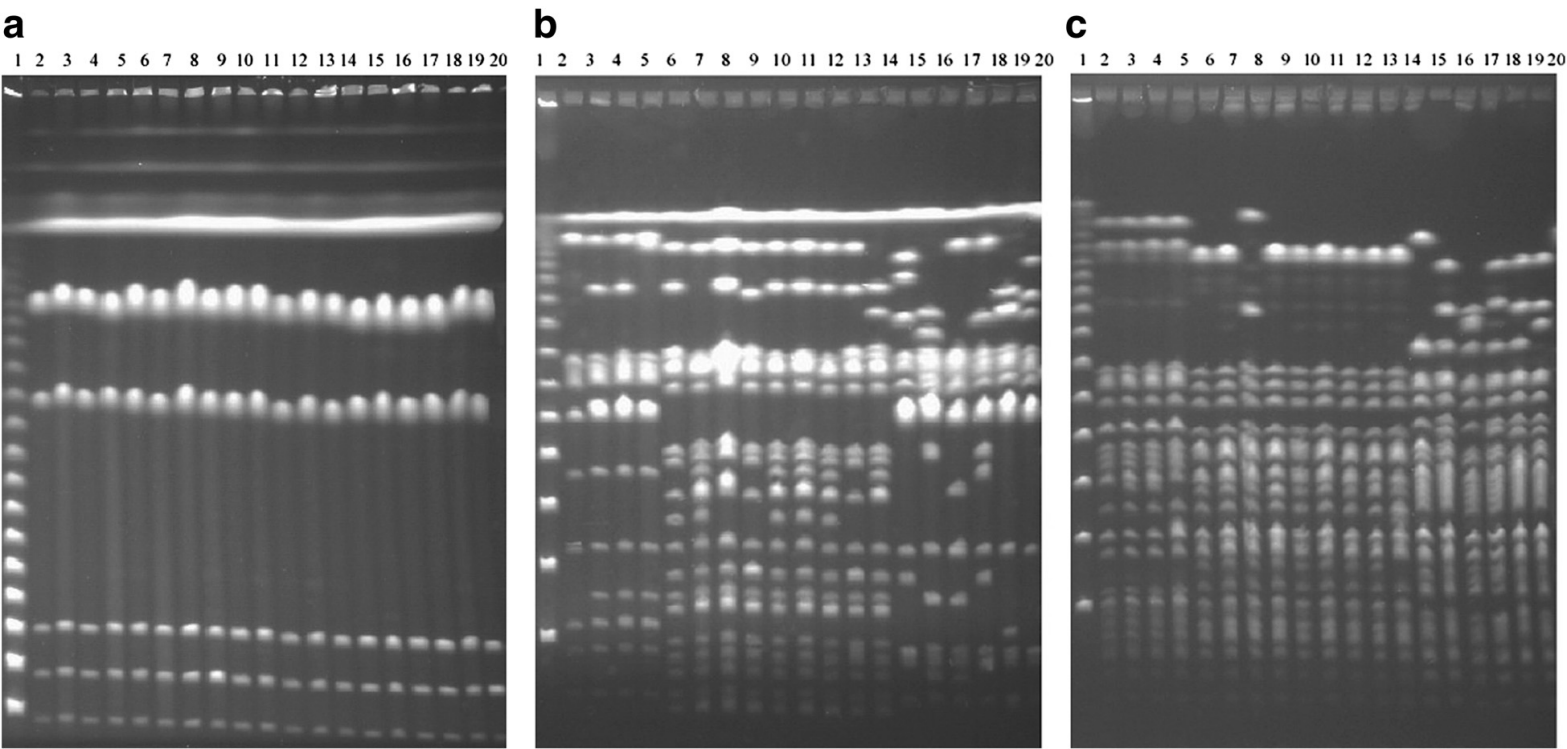
**Genomic DNA PFGE patterns of representative *Salmonella *strains, cleaved with I-CeuI (a), XbaI (b) and SpeI (c), respectively.** Lanes: 1, DNA size Marker; 2-5, S. *enteritidis* (SE310, SE154, SE301, LK5); 6-14, *S. pullorum* (RKS5078, CDC1983-67, SARB51, 04–6767, NS387, 00–19557, 02–15951, 03–16062, 98–13777); 15–20, *S. gallinarum* (287/91, RKS5021, SGSC2293, 91–29327, 90–5289, 92–7995).

As there were only 26 sequenced *Salmonella* genomes available for this study and, more importantly, most of the thirteen lineages had only one strain sequenced, we needed to confirm the genomic homogeneity within individual lineages and the genomic distinction across different lineages by looking at larger numbers of wild type strains. As conservative endonuclease cleavage sites may reflect phylogenetic relationships of bacteria [8,19], we carried out comparative analysis of representative *Salmonella* lineages by the pulsed field gel electrophoresis (PFGE) techniques on strains of *S. enteritidis, S. pullorum* and *S. gallinarum* isolated at broad ranges of time or geographic localities. Consistent with previous findings, the endonuclease I-CeuI revealed indistinguishable cleavage patterns among the *Salmonella* lineages (Figure 3a). On the other hand, the endonucleases XbaI and SpeI revealed cleavage patterns that are common to strains of the same *Salmonella* lineage and distinct among different *Salmonella* lineages (Figure 3b & c). Since these three *Salmonella* lineages are of the most closely related among all *Salmonella* lineages so far analyzed, the genomic distinction among them strongly indicates the existence of genetic boundaries between them.

### Genetic boundaries in *Yersinia* and *Staphylococcus*

**Figure 4:**
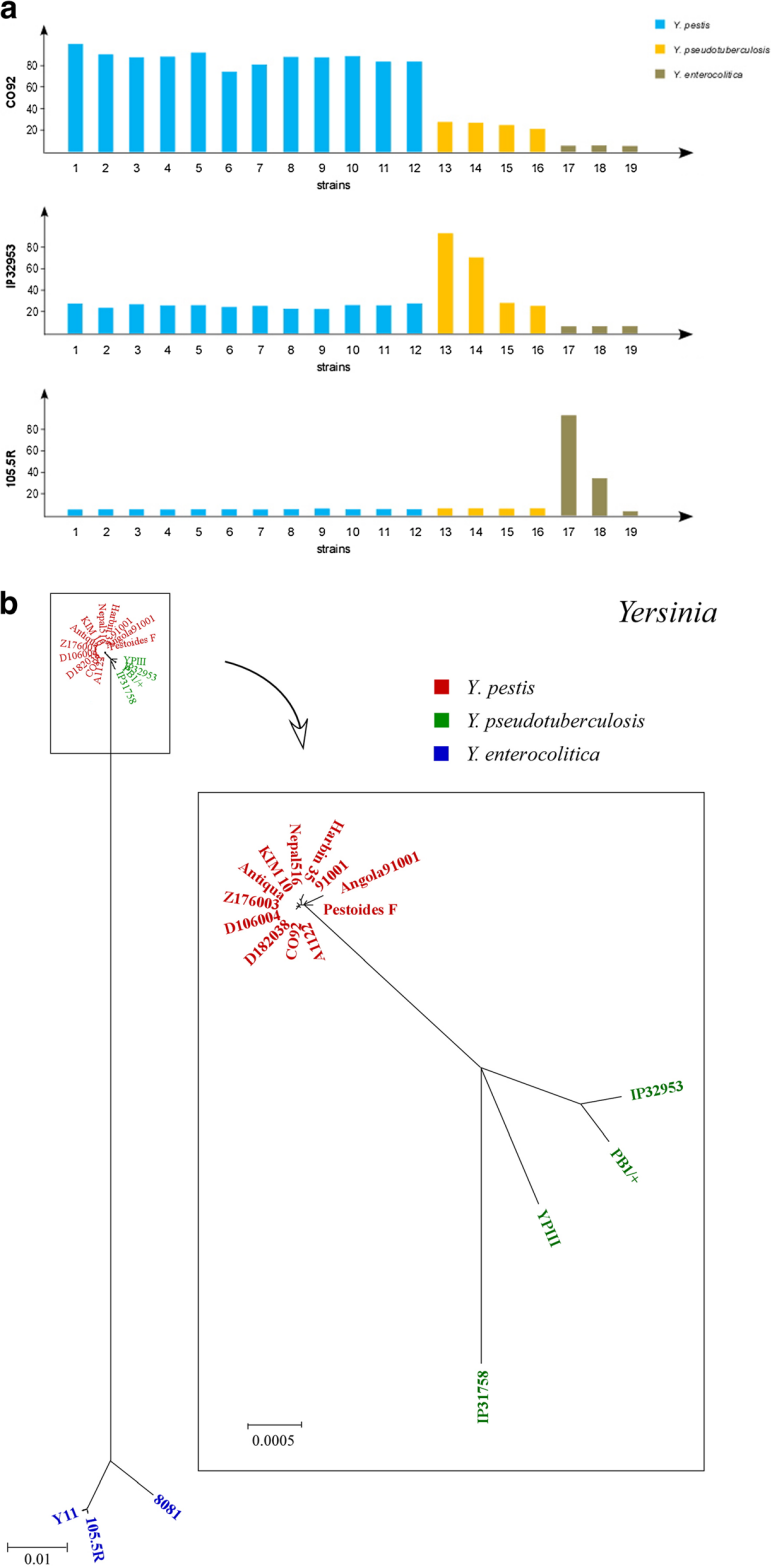
**Clustering of *Yersinia *lineages. a**, Genomic comparison among the *Yersinia* strains. Strains: 1–12, *Y. pestis* (CO92, Z176003, KIM 10, Antiqua, A1122, Angola, Pestoides F, D106004, D182038, Nepal516, Harbin 35, and 91001); 13–16, *Y. pseudotuberculosis* (IP 32953, PB1/+, YPIII, and IP 31758); 17 & 18, *Y. enterocolitica* subsp. *palearctica* (105.5R and Y11); and 19, *Y. enterocolitica* subsp. *enterocolitica* 8081. Upper panel: *Y. pestis* CO92 genome as the target, with all the other 18 genomes compared to it; middle panel: *Y. pseudotuberculosis* IP 32953 genome as the target, with all the other 18 genomes compared to it; lower panel: *Y. enterocolitica* subsp. *palearctica* 105.5R genome as the target, with all the other 18 genomes compared to it. **b**, Phylogenetic tree for the *Yersinia* strains based on conserved sequences that are concatenated and aligned for tree construction.

Having obtained results from *Salmonella* analysis that supported the hypothesis of genetic boundary, we wanted to know if findings from *Salmonella* could be generalized in other bacteria. We chose bacteria from two representative genera, including *Yersinia*, which is closely related to *Salmonella*, and *Staphylococcus*, which is very distantly elated to *Salmonella*. The 19 *Yersinia* strains compared in this study included one of *Yersinia enterocolitica* subsp. *enterocolitica*, two of *Y. enterocolitica* subsp. *palearctica*, four of *Y. pseudotuberculosis*, and twelve of *Y. pestis* (Additional file 1: Table S1). All 12 *Y. pestis* strains had most of their genes in common (Additional file 7: Table S7) and shared high genomic homogeneity (76% or more of their common genes had 100% sequence identity; Additional file 8: Table S8), a situation that is very similar to a *Salmonella* lineage such as *S. typhimurium*; strains of other *Yersinia* lineages had abruptly lower percentages of common genes and genes sharing 100% sequence identity when compared to *Y. pestis* (Additional file 7: Tables S7 and Additional file 8: Table S8 and Figure 4a). Phylogenetic studies also supported the genetic boundary hypothesis (Figure 4b).

**Figure 5:**
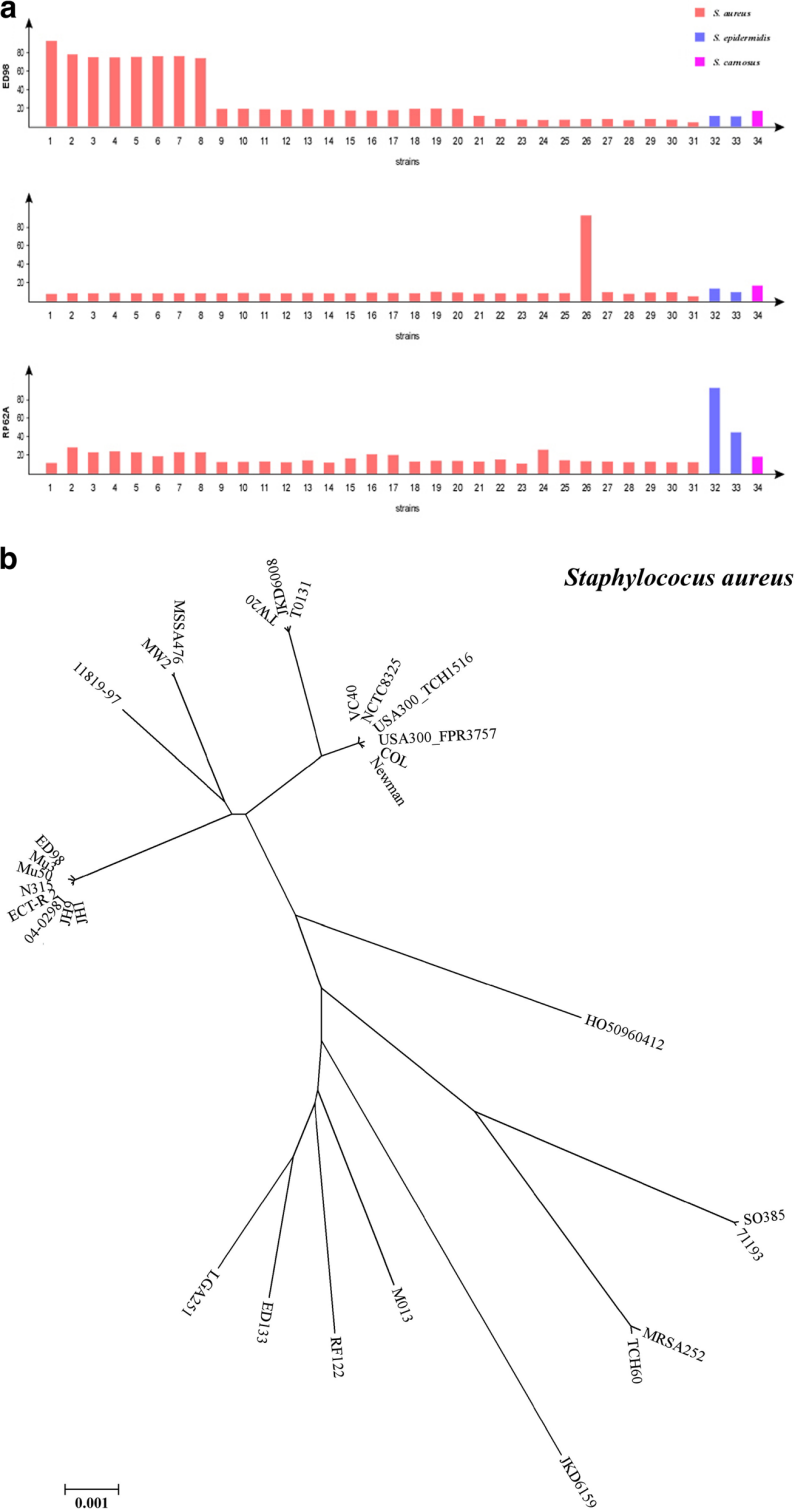
**Clustering of *Staphyococcus *lineages. a**, Genomic comparison among the *Staphyococcus* strains. Strains: 1–31, *S. aureus* (ED98, N315, Mu50, JH1, Mu3, ECT-R 2, 04–02981, JH9, VC40, NCTC8325, USA300_TCH1516, USA300_FPR3757, COL, Newman, TW20, JKD6008, T0131, 11819–97, MW2, MSSA476, HO 5096 0412, S0385, 71193, MRSA252, TCH60, JKD6159, M013, RF122, ED133, LGA251, MSHR1132); 32 & 33, *S. epidermidis* (RP62A, ATCC 12228); and 34, *S. carnosus* TM300. Upper panel: *S. aureus* ED98 genome as the target, with all the other 33 genomes compared to it; middle panel: *S. aureus* JKD6159 genome as the target, with all the other 33 genomes compared to it; lower panel: *S. epidermidis* RP62A genome as the target, with all the other 33 genomes compared to it. **b**, Phylogenetic tree for the *S. aureus* strains based on conserved sequences that are concatenated and aligned for tree construction. Some strains form clusters as tightly as those of *Salmonella* lineages or *Y. pestis*, suggesting a taxonomic species of *Staphyococcus* may actually contain multiple natural species, each being as cohesive as a *Salmonella* lineage.

We also compared *Staphylococcus* strains (Additional file 1: Table S1). The 34 strains were isolated from different regions of the world, including 31 *S. aureus* strains, two *S. epidermidis* strains and one *S. carnosus* strain. The *Staphylococcus* strains had much more divergence from one another than those of *Salmonella* or *Yersinia*; within *S. aureus,* the divergence was also much greater than that of *Salmonella*, However, some of the *S. aureus* strains did cluster together as tightly as those of *S. typhimurium* (Additional file 9: Table S9 and Additional file 10: Table S10; Figure 5). This finding indicates that natural species of bacteria like *Staphylococcus* that are well known to be genetically very diverse may actually be as cohesive as those of *Salmonella*, implying that the name *S. aureus* may actually contain many distinct natural species with genetic boundaries clearly and digitally “visible” among them.

## Discussion

This study aims at one key question: do bacteria exist as discrete clusters or do they spread all over continuously to span the whole phylogenetic spectrum or, asked in another way, do bacteria exist as natural species that are isolated by genetic boundaries into discrete phylogenetic clusters? This question is central to bacterial systematics or, in a sense, to biology, but so far there was no evidence-based answer or experimentally testable hypothesis. As a result, bacteria have been classified into species by largely arbitrary cut-offs at 70% DNA-DNA association and 97% 16S rRNA sequence identity. Therefore, genera, families and higher taxonomic ranks based on the arbitrary species can only be arbitrary, reflecting not necessarily accurate natural relationships among the bacteria. Through this study, we show that genetic boundaries circumscribing bacteria are objective and can be described digitally. Specifically, *Salmonella* lineages, such as *S. typhi, S. typhimurium, S. gallinarum* and *S. pullorum* analyzed in this study, may be defined as species, since clear-cut genetic boundaries have been unambiguously demonstrated among them. We also demonstrated the existence of similar clear-cut genetic boundaries in other bacteria exemplified by *Yersinia* and *Staphylococcus*.

The first line of evidence indicating the existence of genetic boundaries isolating bacteria into discrete phylogenetic clusters was in fact provided by physical analyses of bacterial genomes with the PFGE techniques. For example, the cleavage sites of certain endonucleases such as XbaI and SpeI are highly conservative within a *Salmonella* lineage [29]; of great significance, the conservation of cleavage sites disappear abruptly across the lineages, even between those as closely related as *S. pullorum* and *S. gallinarum* (see Figure 3). A plausible explanation for the genomic conservation within a bacterial lineage (to be defined as natural species) is that bacteria of a species occupy a niche not congruent with those of bacteria in other species; a subpopulation of this species may become dominant in the niche and purge other subpopulations of the same species, retaining a genome structure representative of the extant species. Phylogenetic analysis shows that strains of the same *Salmonella* lineage cluster very tightly together and different *Salmonella* lineages are clearly isolated with certain evolutionary distances on the genealogical tree as a result of independent accumulation of nucleotide variations over long evolutionary times.

To look into the molecular basis of the genetic boundaries isolating bacterial into natural clusters (i.e., species), we carried out systematic comparisons of the sequenced *Salmonella* genomes and found a sharp drop in the number of genes that have 100% sequence identity across the *Salmonella* lineages, a finding that further demonstrates the rather rigid genetic isolation among the *Salmonella* lineages. Genetic isolation between *Salmonella* lineages were also demonstrated by reduced recombination efficiency between *S. gallinarum* and *S. pullorum*, which are the most closely related *Salmonella* lineages so far known. We postulated the existence of genetic barriers among different *Salmonella* lineages, but the molecular basis is still largely unknown. Although the mismatch repair system may account for at least part of the genetic boundaries as a kind of barriers against gene flow between bacteria of different species [9,30-32], obviously the genetic boundaries are formed by multiple factors, many of which are yet to be identified.

## Conclusion

We conclude that bacteria exist as discrete biological clusters to be called natural (rather than arbitrary) species; the genetic boundaries separating the bacterial species can be described digitally and may be used for the establishment of objective and universal criteria to define bacterial species. *Salmonella* lineages are separated by clear-cut genetic boundaries and therefore each should be re-classified as species. Our method requires whole genome sequences from only representative bacterial strains and most others can be compared by PFGE analyses.

## Methods

### Bacterial strains and phages

Information on the *Salmonella* strains used in this study can be found at the *Salmonella* Genetic Stock Center (http://www.ucalgary.ca/~kesander/). Bacteriophage P22 (HT105/1 int-201) was routinely grown on *S. typhimurium* LT2 and was used in the transduction experiments.

### Enzymes and chemicals

I-CeuI, XbaI and SpeI were purchased from New England Biolabs, and proteinase K was from Roche. The other reagents were mainly from Sigma.

### PFGE analyses of genomic DNA

Bacterial genomic DNA isolation, endonuclease cleavage with I-CeuI, XbaI and SpeI, and separation of the cleavage fragments by PFGE were as described previously [7,19,25].

### Bacteriophage-mediated transduction experiments

P22 mediated transduction methods were as described [25,33] to move DNA between different *Salmonella* strains.

### Genomic and statistics analysis tools

Phylogenetic tree construction was done with MEGA4.0.2 and CLUSTALW. The statistical analyses of transduction data were performed by using software SPSS v20.

## Competing interests

The authors declare that they have no competing interests.

## Authors’ contributions

LT carried out the experimental work and genomic sequence analysis, co-supervised YL and XD, both of whom participated in the experimental work, and determined the molecular basis of the genetic boundaries. RNJ and GRL participated in data analysis. SLL conceived of and designed the study and wrote the manuscript. All authors read and approved the final manuscript.

## Supplementary Material

Additional file 1: Table S1 Genomes analyzed in this study. Click here for file

Additional file 2: Table S2 Numbers of genes in common between pairs of *Salmonella* strains. Click here for file

Additional file 3: Table S3 Numbers of genes with 100% sequence identity between pairs of *Salmonella* strains. Click here for file

Additional file 4: Table S4 Percentages of common genes with 100% sequence identity between pairs of *Salmonella* strains. Click here for file

Additional file 5: Table S5 Number of transductants resulting from P22-mediated transfer moving the *ompD*159 DNA between *S. pullorum* and *S. gallinarum*. Click here for file

Additional file 6: Table S6 Number of P22-mediated transductants between *S. gallinarum* and *S. pullorum*. Click here for file

Additional file 7: Table S7 Numbers of genes in common between pairs of *Yersinia* strains. Click here for file

Additional file 8: Table S8 Percentages of common genes with 100% sequence identity between pairs of *Yersinia* strains. Click here for file

Additional file 9: Table S9 Numbers of genes in common between pairs of *Staphylococcus* strains. Click here for file

Additional file 10: Table S10 Percentages of common genes with 100% sequence identity between pairs of *Staphylococcus* strains. Click here for file
