# Supplementary material for: Defining natural species of bacteria: clear-cut genomic boundaries revealed by a turning point in nucleotide sequence divergence
Source: BMC Genomics. 2013 Jul 18;14:489. doi: 10.1186/1471-2164-14-489 (PMC3751360; doi:10.1186/1471-2164-14-489)
Supplement: Additional file 1: Table S1 — Genomes analyzed in this study. [file 1471-2164-14-489-S1.doc]

**Supplementary Table S1. Genomes analyzed in this study.**

| **Lineage** | **Strain No.** | **Accession No.** |
| --- | --- | --- |
| ***S. typhimurium*** | LT2 | AE006468 |
|  | 14028S | CP001363 |
|  | SL1344 | FQ312003 |
|  | D23580 | FN424405 |
|  | ST4/74 | CP002487 |
|  | UK/1 | CP002614 |
| ***S. typhi*** | Ty2 | AE014613 |
|  | CT18 | NC_003198 |
|  | P-stx-12 | NC_016832 |
| ***S. paratyphi* A** | ATCC9150 | CP000026 |
|  | AKU_12601 | FM200053 |
| ***S. pullorum*** | RKS5078 | CP003047 |
|  | CDC1983-67 | CP003786.1 |
| ***S. gallinarum*** | 287_91 | AM933173 |
|  | SG9 | CM001153 |
| ***S. enteritidis*** | P125109 | AM933172 |
| ***S. choleraesuis*** | A50 | CM001062 |
|  | SC-B67 | AE017220 |
| ***S. paratyphi* C** | RKS4594 | CP000857 |
| ***S. heidelberg*** | B182 | NC_017623 |
|  | SL476 | CP001120 |
| ***S. dublin*** | CT_02021853 | CP001144 |
|  | SD3246 | CM001151 |
| ***S. newport*** | SL254 | CP001113 |
| ***S. schwarzengrund*** | CVM19633 | CP001127 |
| ***S. agona*** | SL483 | CP001138 |
|  |  |  |
| ***Yersinia pestis*** | CO92 | NC_003143 |
|  | Z176003 | NC_014029 |
|  | KIM 10 | NC_004088 |
|  | Antiqua | NC_008150 |
|  | A1122 | NC_017168 |
|  | Angola | NC_010159 |
|  | Pestoides F | NC_009381 |
|  | D106004 | NC_017154 |
|  | D182038 | NC_017160 |
|  | Nepal516 | NC_008149 |
|  | Harbin 35 | NC_017265 |
|  | 91001 | NC_005810 |
| ***Yersinia pseudotuberculosis*** | IP 32953 | NC_006155 |
|  | PB1/+ | NC_010634 |
|  | YPIII | NC_010465 |
|  | IP 31758 | NC_009708 |
| ***Yersinia enterocolitica subsp. palearctica*** | 105.5R | NC_015224 |
|  | Y11 | NC_017564 |
| ***Yersinia enterocolitica subsp. enterocolitica*** | 8081 | NC_008800 |
|  |  |  |
| ***Staphylococcus aureus*** | ED98 | NC_013450 |
|  | N315 | NC_002745 |
|  | Mu50 | NC_002758 |
|  | JH1 | NC_009632 |
|  | Mu3 | NC_009782 |
|  | ECT-R 2 | NC_017343 |
|  | 04-02981 | NC_017340 |
|  | JH9 | NC_009487 |
|  | VC40 | NC_016912 |
|  | NCTC 8325 | NC_007795 |
|  | USA300_TCH1516 | NC_010079 |
|  | USA300_FPR3757 | NC_007793 |
|  | COL | NC_002951 |
|  | Newman | NC_009641 |
|  | TW20 | NC_017331 |
|  | JKD6008 | NC_017341 |
|  | T0131 | NC_017347 |
|  | 11819-97 | NC_017351 |
|  | MW2 | NC_003923 |
|  | MSSA476 | NC_002953 |
|  | HO 5096 0412 | NC_017763 |
|  | S0385 | NC_017333 |
|  | 71193 | NC_017673 |
|  | MRSA252 | NC_002952 |
|  | TCH60 | NC_017342 |
|  | JKD6159 | NC_017338 |
|  | M013 | NC_016928 |
|  | RF122 | NC_007622 |
|  | ED133 | NC_017337 |
|  | LGA251 | NC_017349 |
|  | MSHR1132 | NC_016941 |
| ***Staphylococcus epidermidis*** | RP62A | NC_002976 |
|  | ATCC 12228 | NC_004461 |
| ***Staphylococcus carnosus*** | TM300 | NC_012121 |
